# An Automated Image Analysis System to Measure and Count Organisms in Laboratory Microcosms

**DOI:** 10.1371/journal.pone.0064387

**Published:** 2013-05-29

**Authors:** François Mallard, Vincent Le Bourlot, Thomas Tully

**Affiliations:** 1 CNRS/UPMC/ENS, Écologie et Évolution, UMR 7625, École Normale Supérieure, Paris, France; 2 CERES - ERTI, École Normale Supérieure, Paris, France; 3 ESPE de Paris, Université Paris 4 - Sorbonne, Paris, France; National Cancer Institute, National Institutes of Health, United States of America

## Abstract

1. Because of recent technological improvements in the way computer and digital camera perform, the potential use of imaging for contributing to the study of communities, populations or individuals in laboratory microcosms has risen enormously. However its limited use is due to difficulties in the automation of image analysis. 2. We present an accurate and flexible method of image analysis for detecting, counting and measuring moving particles on a fixed but heterogeneous substrate. This method has been specifically designed to follow individuals, or entire populations, in experimental laboratory microcosms. It can be used in other applications. 3. The method consists in comparing multiple pictures of the same experimental microcosm in order to generate an image of the fixed background. This background is then used to extract, measure and count the moving organisms, leaving out the fixed background and the motionless or dead individuals. 4. We provide different examples (springtails, ants, nematodes, daphnia) to show that this non intrusive method is efficient at detecting organisms under a wide variety of conditions even on faintly contrasted and heterogeneous substrates. 5. The repeatability and reliability of this method has been assessed using experimental populations of the Collembola *Folsomia candida*. 6. We present an ImageJ plugin to automate the analysis of digital pictures of laboratory microcosms. The plugin automates the successive steps of the analysis and recursively analyses multiple sets of images, rapidly producing measurements from a large number of replicated microcosms.

## Introduction

Because of their relatively short generation time and ease of rearing, invertebrate species are ideal for studying population dynamics and life history traits: *Daphnia* [1], [2], *Drosophila* [3], mites [4], Collembola [5], [6], Nematodes [7], [8]. But even in these convenient model organisms, data collection is often made by eye which is possible when populations are very small [6], [9], but can soon become far too time consuming.

Measurements can be made using digital images of individuals or populations. Pictures are ideal because they are taken rapidly, they are innocuous, cheap and can be stored and re-observed if necessary. During the past fifteen years, the technological improvements of digital cameras, hard drive storage capacities and computer performances [10] has radically changed the way researchers use images to collect and store information on their experiments. Numerous image analysis software are now available [11] many of which are open-source [12]. In experimental ecology, such progresses enables the acquisition of large amounts of pictures and the tracking of individual behaviour, fecundity or growth trajectory on a fine time scale and over long periods of time. The measurement itself can be made manually on a computer using appropriate image analysis software such as ImageJ [12], [13] to estimate egg and body sizes for instance [5], [14]. Massive image capture and analysis can also be used to follow the size and structure of an experimental population. But even on pictures, measurements remain time-consuming and may quickly become impractical. Reliable and reproducible automatic counting and measuring methods are then needed.

Various authors have designed and proposed image analysis methods to automatically measure or count small organisms in the laboratory [15]–[19]. But these methods fail at dealing with heterogeneous substrates and the particles and dead individuals that are prone to be detected as living individuals in the automatic census. Here we present a simple image processing method that can increase the efficiency and reliability of particle detection and population monitoring which has seldom been used before in ecology [20], [21]. The principle used here is based on the particle analysis developed in the ImageJ multi-tracker plugin [22]. The idea is to compare several pictures of the studied microcosm to construct a composite picture made up from the motionless elements. This will generate a background image which can then be removed from the original pictures to show only the elements that moved, which are often the organisms being studied. It requires simple material (a digital camera or a webcam, a stand and a good lighting device) and an image processing program. Here we used the open-source ImageJ software [13].

We first describe the different steps of the image segmentation using a laboratory population of the springtail *Folsomia candida* (Collembola, Isotomidae) as a model microcosm [23]. The same method is applied using different environments to illustrate the variety of biological models and questions that can be tackled with it. We then give some quantitative assessments of the method’s reliability and robustness using again the collembolans as a case study. Finally, we describe an ImageJ plugin that we have developed to automate batch laboratory population census.

## Materials and Methods

### Method Overview

#### Image acquisition

**Figure 1:**
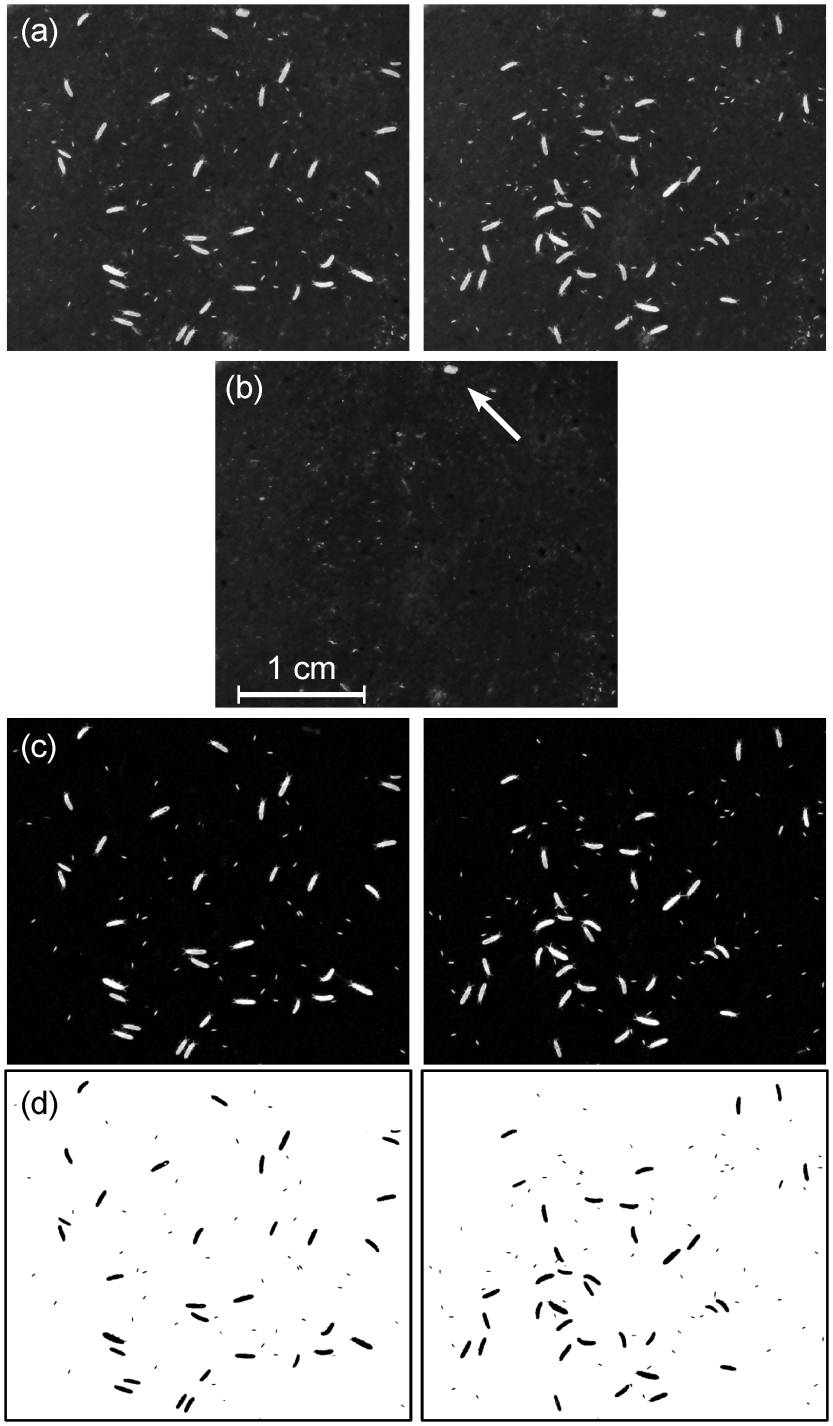
The successive steps of the image analysis. (a) Two of the original pictures of a population of Collembola *Folsomia candida* raised on humid plaster of Paris darkened with Indian ink. (b) The constructed still background picture. Each pixel is calculated as the darker pixel of the original set of pictures. (c) Subtracting (b) from (a) removes the still background and reveals the springtails. The white grain in the background (see arrow) has disappeared. (d) After thresholding it becomes possible to count and measure the springtails with great confidence.

The first step is to take a set of pictures (usually from three to five) of the microcosm being studied with constant framing and lighting (Figure 1a). The camera and the microcosm have to be kept in exactly the same position while the stack of pictures are taken. We used a digital camera fixed on a stand and remotely controlled by a computer (Nikon D300 and CameraControlPro). Constant, homogeneous and strong lighting was provided by four LED bulbs (Pikaline, 16W, 650 lumens). It is always better to have a good light homogeneity but, as discussed below, the method can compensate for partial lighting inhomogeneity. Temporal stability of lighting conditions between pictures in a stack is more important. Using fluorescent lamps is not recommended since their light intensity varies, causing lighting heterogeneity between pictures. We also recommend not to use incandescent light bulbs as they produce a lot of heat that can perturb or harm the studied organisms.

#### Creation of a background picture

Using ImageJ, the pictures are put into a stack (menu *File, Import*, *Image Sequence*) and then compared (*Image*, *Stacks, Zproject*) to generate a new image composed of all the elements that remained motionless (Figure 1b). Each pixel in the stack is analysed and at each position only the one of minimal intensity (i. e. the darkest one) is kept. Other methods (median, mean) are recommended if the picture quality is heterogeneous or if the pictures are taken on a time scale long enough for the lighting conditions to vary slowly. The resulting image - the still background - is then subtracted from each of the original stack pictures (*Process*, *Image Calculator*, Figure 1). This produces a new stack of images that only contains the mobile elements, here the collembolans (Figure 1c).

#### Detecting, measuring and counting the organisms

The next step is the thresholding procedure that will transform the 8-bit images (256 grey levels) into black and white 2-bit images (*Image*, *Adjust*, *Threshold*, Figure 1c). The selected threshold value determines the grey level above which the pixels will become white and under which they will become black and be measured. Removing the substrate image created a homogeneous background, on which the organisms are clearly visible. Particle measurement becomes less sensitive to the chosen threshold value, since a large range of threshold values gives equivalent results. Once the 2-bit pictures are created, the ImageJ “*Analyse Particles*” function can be used to count and measure the particles.

### Application Examples from Various Biological Systems

To prove the wide potential use of this method in experimental ecology, we worked on several other biological systems. In the first two, we counted and measured other model organisms in their usual laboratory environments (nematodes in agar plate and zooplankton in a pond sample). We then give two other methodological applications: the tracking of a single collembolan and the measurement of ants’ activity during a long period of time. No endangered or protected species were involved and no specific permission was required. The animals were just briefly used for taking a set of pictures without being harmed by handling them.

#### Detecting and counting nematodes in a petri dish

**Figure 2:**
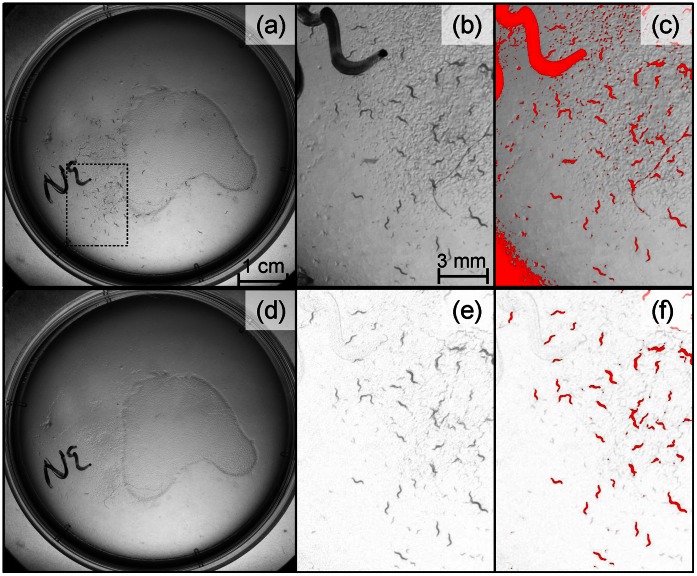
Detecting nematodes on agar in a Petri dish. Several pictures of a standard nematode rearing plate such as (a) have been taken. From this stack of pictures, a background image (d) has been created. The four other panels are close-up views of the same area (dashed rectangle of (a)) before (b, c) and after (e,f) the removal of the background image. A standard particle analysis run on the original pictures (b,c) failed to bring out only the nematodes: an optimal thresholding is impossible because of the background heterogeneity (c). But removing the still background (e) allows a much more efficient detection of the nematodes (f).

This method was tested for detecting nematodes (*Caenorhabditis elegans*) on agar in a Petri dish (Figure 2a). We took a set of 10 pictures interspaced at approximately 5 to 10 sec. We used the same camera as previously and the Petri dish were lit up from below using the lighting device of a dissection microscope.

#### Counting and identifying pond copepods and ostracods

**Figure 3:**
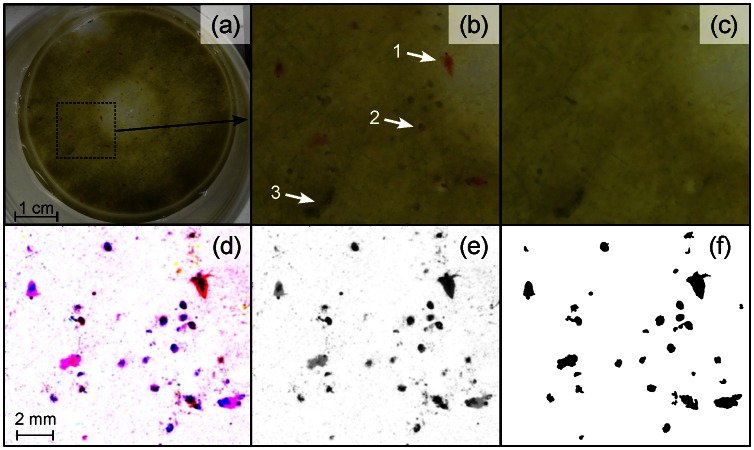
Detecting and counting the zooplankton in a sample of pond water. (a) One picture among five similar ones. (b) A close-up view (dashed rectangle) reveals a few Cladoceran (red *Daphnia*, arrow 1) and several small Ostracods (*Cypris sp.*, arrow 2) on a layer of green algae. The difference between (b) and still background (c) reveals the particles that have moved, i.e. the crustaceans. This picture can be transformed into an 8-bit grey image (e) which can be used to detect, measure and count the zooplankton (f). The immobile dark clumps (arrow 3) are excluded from the census. The colour image (d) can also be used to identify different species.

The method has also been applied for detecting zooplankton (*Daphnia* & *Cypris*) in a sample of pond water containing some filamentous algae (Figure 3a,b). We used the same camera and lighting unit as in the case of the collembolan population. In order to generate the background image five coloured pictures (interspaced at app. 5 to 10 sec) have been compared (Figure 3c).

#### Tracking the movements of an isolated collembolan

**Figure 4:**
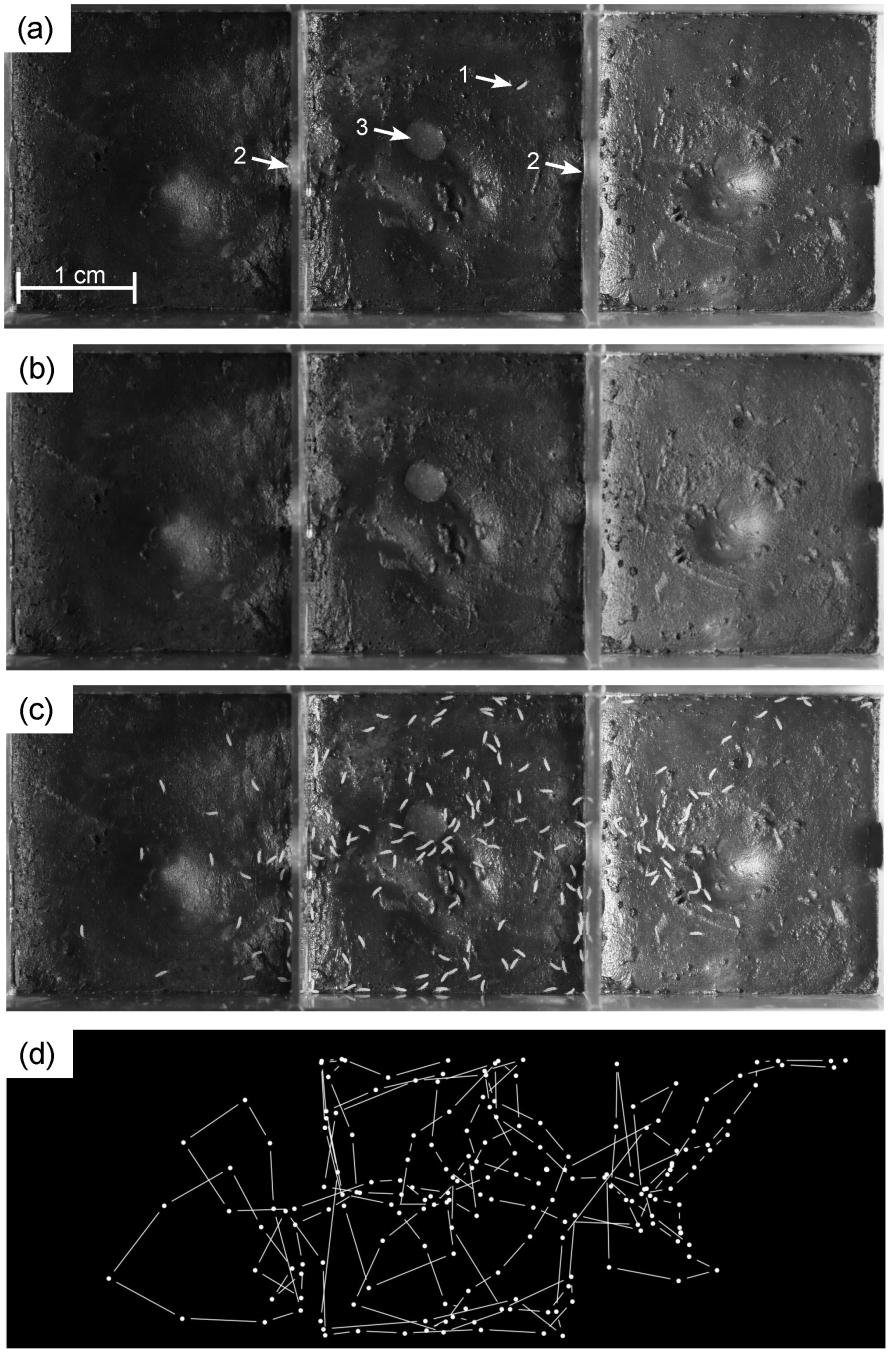
Tracking an isolated collembolan wandering in a container. The container is made of three compartments connected by small holes (arrows 2). A pellet of food (arrow 3) is visible. A picture was taken every 5 sec. during 30 min. The still background (b) was calculated by averaging the 300 pictures. It was then then subtracted from each picture to reveal the collembolan. Picture (c) is the addition of the background (b) and of all the images after the background’s removal. It shows the different positions of the collembola during the follow-up. The full track of the springtail is plotted on panel (d).

In this example, we put a single collembola into a container made of three connected square compartments filled with a substrate of humid and darkened plaster and track its exploratory behaviour (Figure 4). Two hundred pictures of the box were taken, one every 5 sec. The lighting unit used was similar to that previously described for the collembola system.

#### Measuring the temporal dynamics of activity in an ant colony

**Figure 5:**
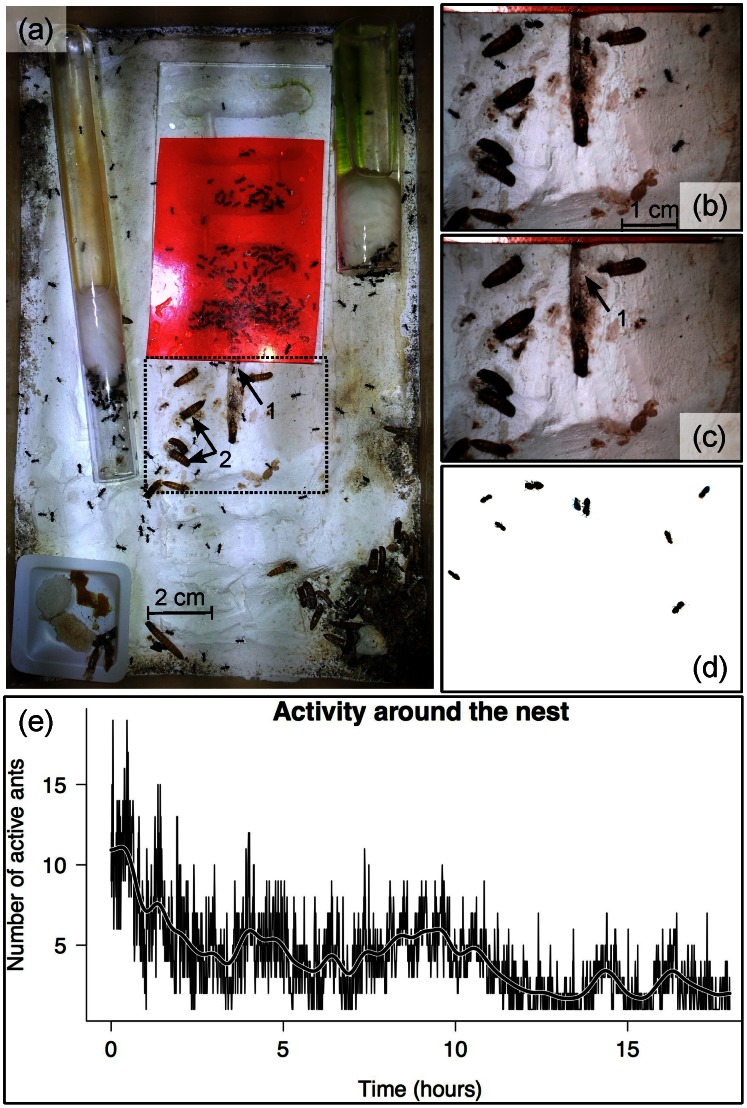
Follow-up of the activity of an ant colony. (a) General view of an ant colony bred in the laboratory on a plaster substrate. The below-ground colony is under the red plastic slate. The ants activity around the nest entrance (arrow 1) has been followed within the dashed rectangle (b). (c) The background image is built up through the comparison of multiple pictures (median value). (d) The difference between (b) and (c) reveals the ants entering and exiting the nest. Immobile dark particles such as remains of food (*Tenebrio* larvae, arrow 2) are discarded from the analysis. (e) By automatically repeating the previous steps, one can easily count the number of active ants around the entrance of the nest. This has been done every 30 sec for 18 h. The graph displays all these measurements and reveals the temporal dynamics of the mean activity around the nest.

A high resolution usb webcam (Dinolite AM7013MZT, 5 Mp) was placed above a laboratory ant colony (Figure 5a) continuously lit by a LED bulb. The camera was programmed to capture an image of the nest entrance (Figure 5, arrow 1) every 30 sec during 18 hours. The ∼2000 images have then been processed with ImageJ in order to compute the fixed background. The number of ants wandering around the nest were then automatically counted on each picture (Figure 5d).

### Method Reliability

#### How many pictures are needed?

**Figure 6:**
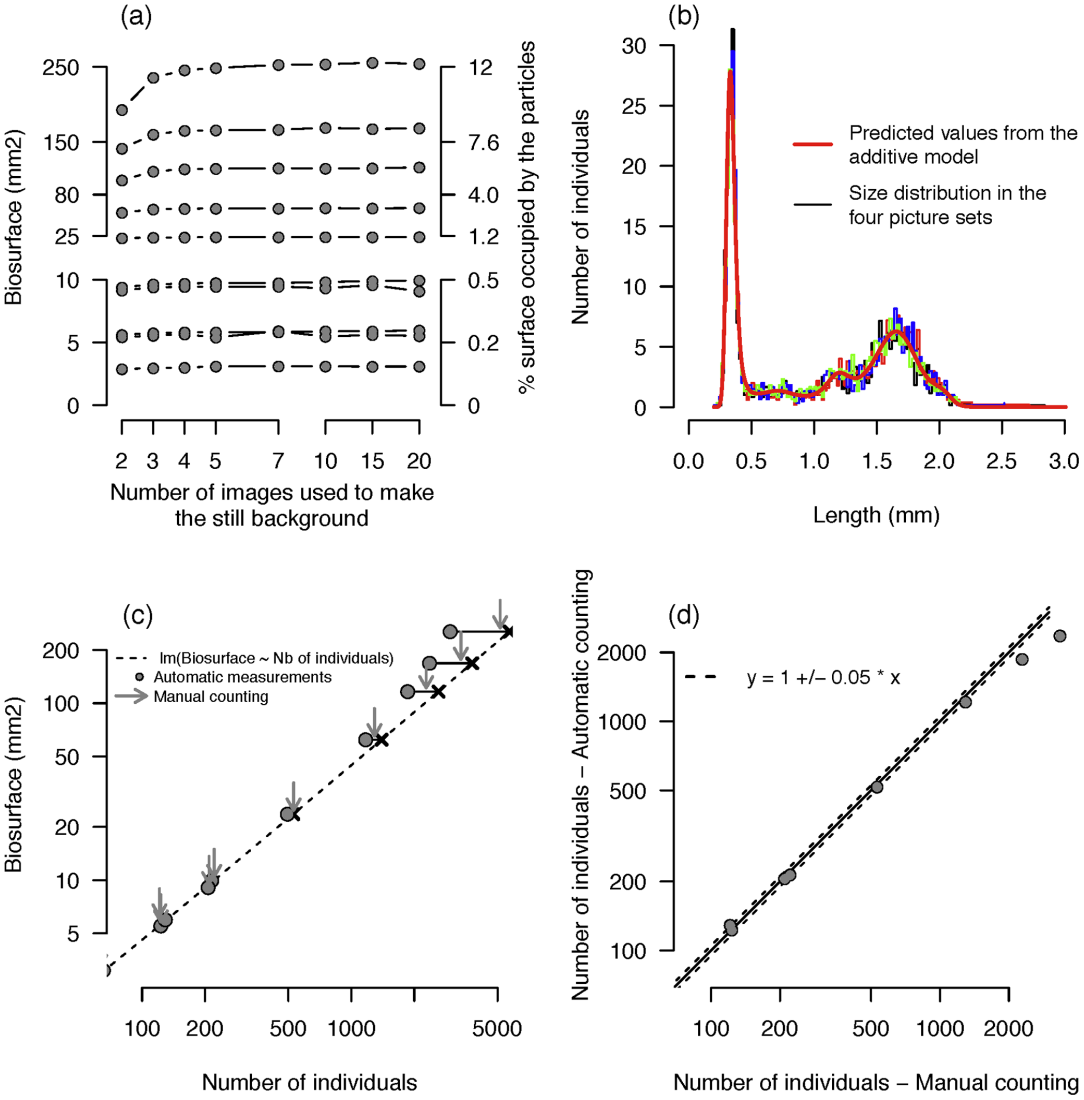
Assessing the method’s reliability on a collembola population. (a) Total measured collembolan biosurface (mm^2^) depending on the number of pictures in the stack. For low densities, two or three pictures are sufficient to have a good and reliable measurement. For higher densities, four or five pictures are needed. (b) Size distribution of the same collembola population. Four different sets of pictures taken by two different users were analysed. The size classes are 0.02 mm width. We fitted a generalised additive model that did not show any significant difference between the pictures nor between the sets. (c–d) Reliability of the automatic counting method. (c) The estimated biosurface measured in 10 boxes and the number of individuals automatically counted (circles). Results of the manual counting are shown (grey arrows) together with a linear regression between the biosurface and the number of individuals calculated for the five lowest density boxes. (d) Comparison of the number of individuals for automatic and manual counting methods.

We studied the minimal number of images needed according to particle density, using springtail populations as an example (Figure 1). We took sets of twenty pictures of ten rearing boxes with increasing densities of juveniles measuring ∼0.15 to 0.5 mm long. For each density, we performed our analysis on different subsets of the whole set of pictures, progressively increasing the number of images used to calculate the background (Figure 6a). A total of 320 sets of pictures were analysed.

#### How repeatable are the measurements of a population structure?

To illustrate the repeatability of the method for measuring a population structure, we analysed four sets of five or six pictures of the same collembolan population (Figure 6b). We divided the size distribution of collembolan (from 0.1 to 3 mm) into 145 classes, each of 0.02 mm width, and analysed the number of individuals in these classes using a generalised additive model (GAM, function *gam* of the *mgcv* library in R) with a Poisson distribution, a cubic regression spline, and an imposed high number of knots (k = 20) to fit the data closely. We then tested differences between the pictures and the stacks of images using anova and Chi-squared tests.

#### Are the counting measurements reliable?

To test whether our counting method is reliable, we compared - for several collembolan densities - the number of particles automatically counted using the background removal method with a measurement made manually. Ten sets of 20 pictures each were analysed automatically to measure the mean number of particles and their total surface. The number of individuals was also measured manually on one picture belonging to each set (Figure 6c–d). We then performed a linear regression between the surface and the number of individuals calculated for the five lowest density boxes. The analyses were all implemented in R 2.15.2 software (http://cran.r-project.org [24]).

### Automated Implementation

We present an ImageJ plugin called BP_sensor for “Batch population sensor” that we have developed to automate the measurements of size and structure in many replicated laboratory populations raised in experimental microcosms (Figure S1). This plugin was specifically designed to track populations of the Collembola *Folsomia candida* but it is versatile enough to be easily adapted to other experimental setups (Figure S2). It automatically performs the recursive census of multiple laboratory populations of collembolans (Figure S3). The code is written in Java and runs within the freely available and open-source ImageJ software [13] (http://rsbweb.nih.gov/ij/). We provide in the Supporting Information the commented Java code (File S2) that can be directly compiled and run with ImageJ as well as a set of image examples (File S3) and some explanations (File S1) to help understand how it works and how it can be customised (see Table S1 in File S1).

## Results and Discussion

### Counting and Tracking Individuals in Various Biological Systems

The method we propose allows a standardisation and automation of microcosm measurements. The segmentation of pictures into regions of interest that match structural units is one of the most critical steps in the process of reducing the complexity of images and extracting information [25]. It usually relies on efficient and precise thresholding. When a single picture is analysed without making use of our background removing procedure, the efficiency of this crucial thresholding step can be improved by (1) controlling the overall luminosity to ensure selecting particles with the same precision everywhere on the picture (homogeneous lighting) and by (2) maximising the contrast between the particles (here living organisms) and their background (here substrate) to get a straight particle segmentation. Removing the motionless background corrects for heterogeneity in lighting conditions and in the underlying substrate. In Figure 4, the blackness of the substrate is heterogeneous on the original pictures which would hinder a standard particle analysis to operate efficiently. This background removal method is also a way to suppress motionless particles and to increase the contrast between moving particles and their substrate (Figure 1c,f). It then becomes possible to automatically adjust the threshold value required for particle measurements since a larger range of the thresholding values will give similar results.

This increased robustness comes at a cost: the multiple pictures have to be perfectly lined up. Even a slight movement can blur the constructed background image and the rest of the analysis will fail. That is why we recommend using a stable stand and a remote shutter release to avoid any movement of the camera. Note that it is possible to translate and re-align images that have moved using the multiplication of the Fourier transformed images (convolution). Our method is also sensitive to temporal luminosity variations: the moving particles can create shadows that darken their surrounding substrate, which locally reduces the efficiency of the background removal. Providing omnidirectional and stable lighting limits the formation of shadows and easily avoids these unwanted effects.

On 8-bit grey images, the background image can either be calculated as the maximum or the minimum grey value in the stack, depending on whether the moving particles are lighter or darker than their substrate. In our case study, the springtails are lighter than the darkened plaster and we used the minimal value. However, using the median or the mean value can be advantageous, especially when the background is very heterogeneous or the contrast between the particles and the substrate is so small that the moving elements can be either darker or lighter than the substrate depending on their positions. For these reasons, we used the mean grey value to calculate the background in all four additional examples. To be efficient, the number of analysed and compared pictures has to be relatively high (at least 4 or 5). Similarly, it is often more efficient to remove the background by computing the “difference” between the original images and the background rather than simply the “subtraction” (see the options of the ImageJ “*Image calculator*” function). Here we applied this method to extract the nematodes from their background as they were either brighter or darker than their agar substrate, depending on the local light reflection.

Given a few adjustments, our measurement method can be applied to various systems. It was quite efficient at highlighting nematodes on agar. Removing the background (Figure 2d) improved the reliability for the detection of nematodes (Figure 2b,c and f) even though the image was faintly contrasted (Figure 2e). Although it improved the detection of adults, the analysis was not perfect since, for example, the small worms were not detected (Figure 2). But the use of a more performant and homogenous lighting unit, combined with some additional image processing such as smoothing, could certainly improve the analysis efficiency.

In the pond water sample (Figure 3), the “substrate” is not motionless: the swimming organisms can shake or move the algae or non living fragments floating around, which can alter the process of background removal. But despite this potential drawback, the method turned out to be pretty efficient at bringing out the zooplankton. It even managed to reveal minor morphological details which were almost hidden in the original pictures (cf. antenna of the *Daphnia* pointed by arrow 1 on Figure 3c). However, long term tracking (as for the ant colony or an isolated springtail) would probably fail owing to too much long term blurring of the background – unless several backgrounds are recursively constructed on a shifting subset of the whole stack of images.

Figure 4 illustrates how this method can be used to easily track the movement and behaviour of an individual exploring an heterogeneous landscape (cf. multi-tracker plugin [22]). The ant activity around the nest is also an interesting application example. We did not track through time a constant number of moving particles nor census a complete population but counted individuals in a partial area of the colony on a long time scale (18 h). The entire data were then grouped on a diagram in order to show the temporal dynamics of the colony’s activity near the nest entrance (Figure 5). We do not compare the results obtained with manual measurements that would be very time consuming. However, the activity measurements are coherent with an initial increased activity, no doubt caused by disturbance during the setting up of the experiment (Figure 5e): the second half of the measurements were done at night and this could explain the decrease of activity.

For both the pond water sample (Figure 3) and the ant colony (Figure 5), the processing was done on coloured pictures. This provided interesting additional information since the colour remained after removal of the background. This information could be used together with the size and shape of the particles to help identify, for instance, different species (here *Daphnia* & *Cypris,* Figure 3d). Such colour images could also benefit from some specific treatment such as decorrelation stretch (DStretch imageJ plugin) to bring out the different particles of interest [26].

### Testing the Method’s Reliability in an Optimised Acquisition System

#### Background calculation - Number of pictures needed

The reliability of the method relies on the quality of the still background image, which has to be free from any moving particle. This will depend on the number of images compared to make the background and on the proportion of the substrate occupied by the creatures. If their density is high, more images are needed for each pixel of the substrate to be visible on at least one image. The replicated analysis performed with increasing number of images in a single stack showed that the total measured biosurface increases with the number of pictures analysed (Figure 6a). But for low densities (0 to 250 individuals - which corresponds to a biosurface of 3 to 10 mm^2^, the rearing box surface measuring 20 cm^2^), this increase is almost negligible. For these low densities, the probability that part of the substrate is covered by a collembola on more than one picture is very low. Taking more than two pictures does not really improve the reliability of the measurements. But for higher densities, three, four or five pictures are needed to reveal the whole background substrate which is needed for reliable and robust estimation of the population biosurface. In our study case, taking more than five pictures never improved the measurement reliability. As a rule, comparing four pictures ensures reliable measurements.

#### Repeatability of a population structure measurement

The estimation of the population structure was repeatable (Figure 6b): the four estimated size distributions were largely overlapping. A fitted generalised additive model to these distributions explains most of the variance (84%) and we found that the estimated size distributions did not differ between the different sets of pictures (χ^2^_3_ = 2.3, p = 0.5), nor between the different pictures within each set (χ^2^_22_ = 23.2, p = 0.4).

#### Reliability of the counting measurement

The reliability of automatic measurements is good for densities below 1000 individuals, but beyond this density the automatic counting underestimates the density (controlled manually): more and more individuals adjoin each other and are then detected as one large particle (Figure 6d). The measured total biosurface then becomes a better proxy for the number of individuals in the box: a projection of the biosurface values of the five highest densities on the linear regression provides a less biased density estimate (Figure 6c). But this correction only works if the individuals have similar size and if they do not overlap, which is the case here. A more complex particle image analysis, like the watershed algorithm [27] could also be used to split up merged individuals.

### Automated Implementation

We have developed an automatic measuring and counting procedure using multiple picture analyses that is easy to use and requires very few calibrations (see ESM). It takes about two hours to obtain five pictures of a hundred populations and to manually sort these pictures in a normed directory tree on the computer. It then takes about one hour for the plugin to analyse these 500 images, count and measure all the individuals in these populations and save the data in distinct files (20 to 30 sec per set of 5 pictures on a 2.5 GHz computer). Altogether about three hours are needed to take a census of one hundred laboratory populations, whose densities can reach a thousand individuals.

One of the major improvements would be to use colour pictures instead of black and white ones. It would not change the background image calculation but would allow more complex segmentation of the particles.

### Conclusion

This simple method is easy to implement and proved to be a useful if not essential image processing step before running a particle detection function. This method is efficient at removing most of the motionless background and to correct for spatially heterogeneous lighting conditions. It is sensitive to even slight movements of the frame or to minor temporal variations in light intensity. It can be used both on grey-level and coloured pictures. It can be applied to many laboratory organisms and to various microcosms. Its implementation has been incorporated into a plugin to automate the analysis of large batches of images, which we hope will help smoothing and accelerate the workflow from microcosm experiments to data analysis.

## Supporting Information

Figure S1 **Example of a directory tree with sorted pictures (upper part) and the resulting tables (lower part).**
(PDF)

Figure S2 **Plugin specification windows.** (a) On this window one is asked to specify the variables described in Table S1 in File S1. If selected, the automatic scaling specification window (b) and the region of interest (roi) selection specifications window (c) will open successively.
(PDF)

Figure S3 **Successive steps of the image processing.** (a) Original picture of a rearing box with its collembola population. A piece of graph paper (arrow 1) and a contrasted black square (arrow 2) can be used as references to scale the measurements. (b) By comparing different images, a background picture is generated: each pixel is the darker pixel of the original set of images. The white moving collembola are automatically discarded. The border of the box (arrow 3) is automatically detected and selected by the plugin. (c) Close-up view of one of the original pictures. Arrow 4 points at a white dirt particle. (d) Before analysing the particles within the region of interest, the plugin removes the background which ensures measuring the moving particles only – motionless white particles or reflections being automatically excluded from the analysis (arrow 5).
(PDF)

File S1 **Some explanations to help understand how the imageJ plugin works and how it can be customised.** Table S1 in this file summarises the different plugin’s options.
(DOC)

File S2 **File S2 comprises our plugin (BP_sensor.java).** The Java code is commented so as to be easily modified. It can be directly compiled and run with ImageJ. File S2 also comprises an other plugin required for our plugin to work smoothly (Wait_For_User.java).
(ZIP)

File S3 **Two sets of pictures of populations of collembolans that can be used as examples to try the plugin.**
(ZIP)
