# Supplementary figures and images for: An Automated Image Analysis System to Measure and Count Organisms in Laboratory Microcosms
Source: PLoS One. 2013 May 29;8(5):e64387. doi: 10.1371/journal.pone.0064387 (PMC3667193; doi:10.1371/journal.pone.0064387)

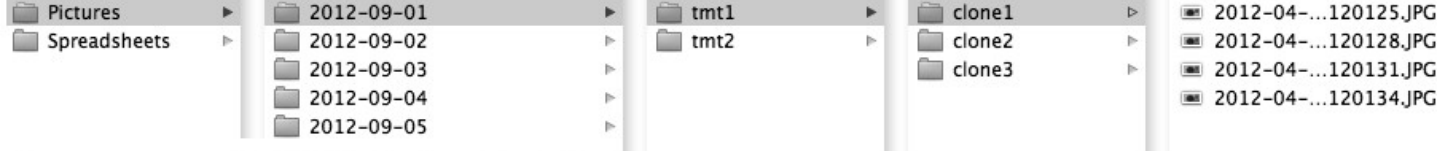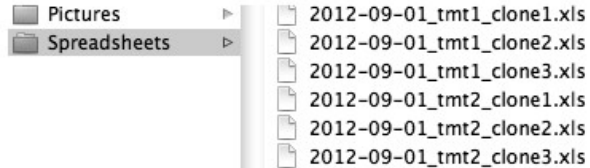

Supplement: Figure S1 — Example of a directory tree with sorted pictures (upper part) and the resulting tables (lower part). (PDF) [file pone.0064387.s001.pdf]

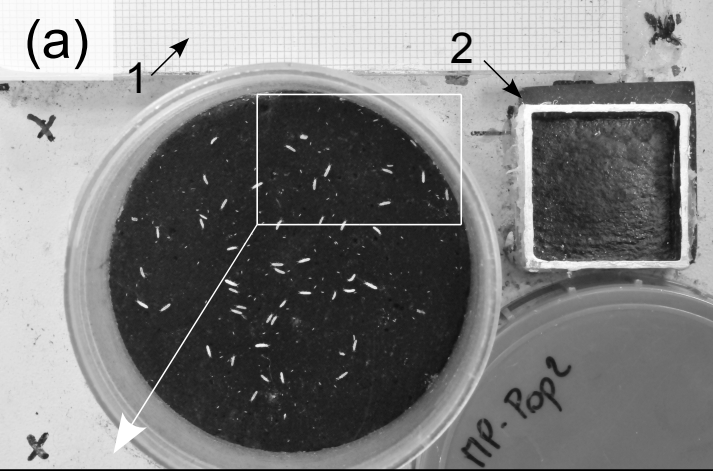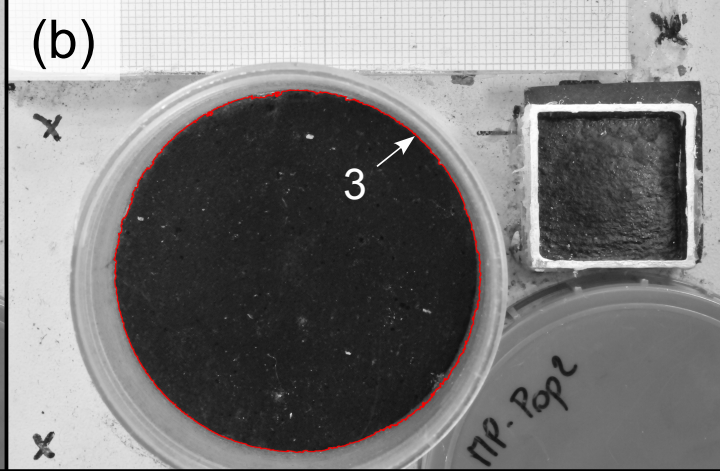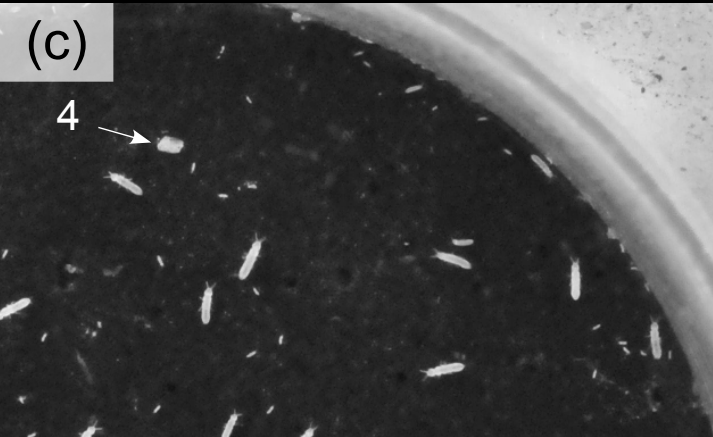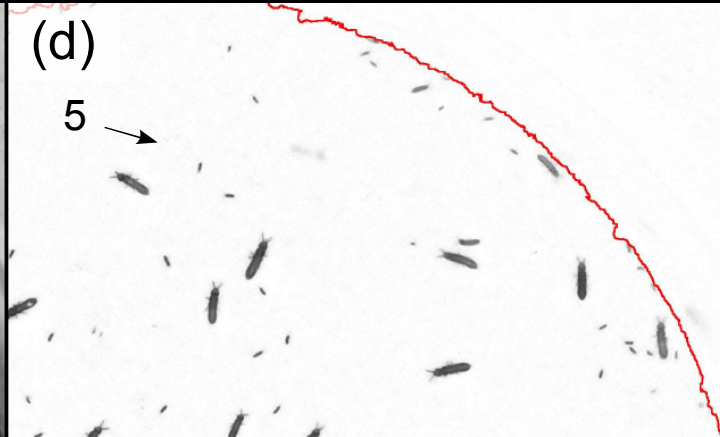

Supplement: Figure S3 — Successive steps of the image processing. (a) Original picture of a rearing box with its collembola population. A piece of graph paper (arrow 1) and a contrasted black square (arrow 2) can be used as references to scale the measurements. (b) By comparing different images, a background picture is generated: each pixel is the darker pixel of the original set of images. The white moving collembola are automatically discarded. The border of the box (arrow 3) is automatically detected and selected by the plugin. (c) Close-up view of one of the original pictures. Arrow 4 points at a white dirt particle. (d) Before analysing the particles within the region of interest, the plugin removes the background which ensures measuring the moving particles only – motionless white particles or reflections being automatically excluded from the analysis (arrow 5). (PDF) [file pone.0064387.s003.pdf]

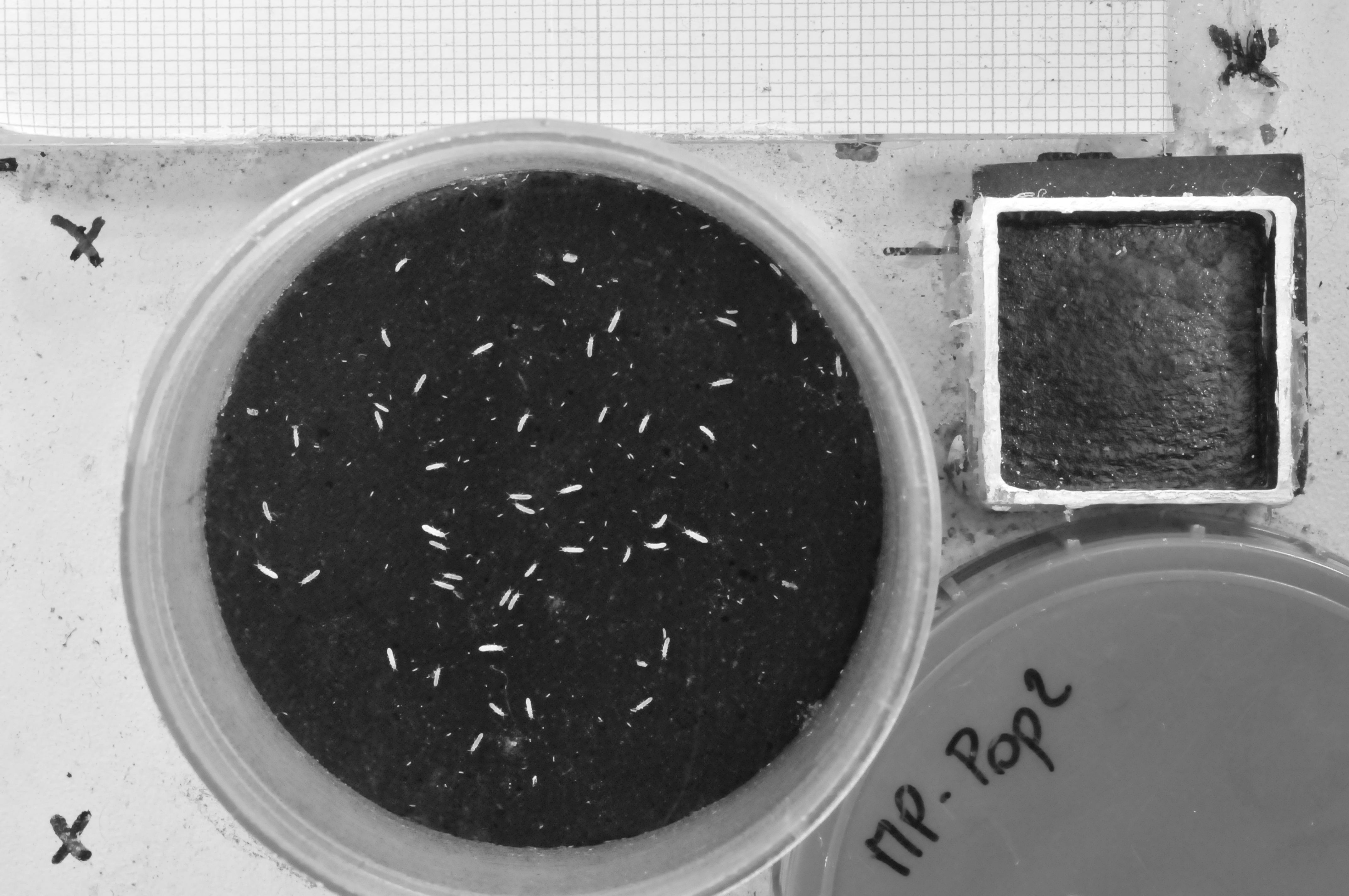

Supplement: File S3 — Two sets of pictures of populations of collembolans that can be used as examples to try the plugin. (ZIP) [file pone.0064387.s006.zip › Picture_set_example1/PR_2011-06-05_181724.jpg]

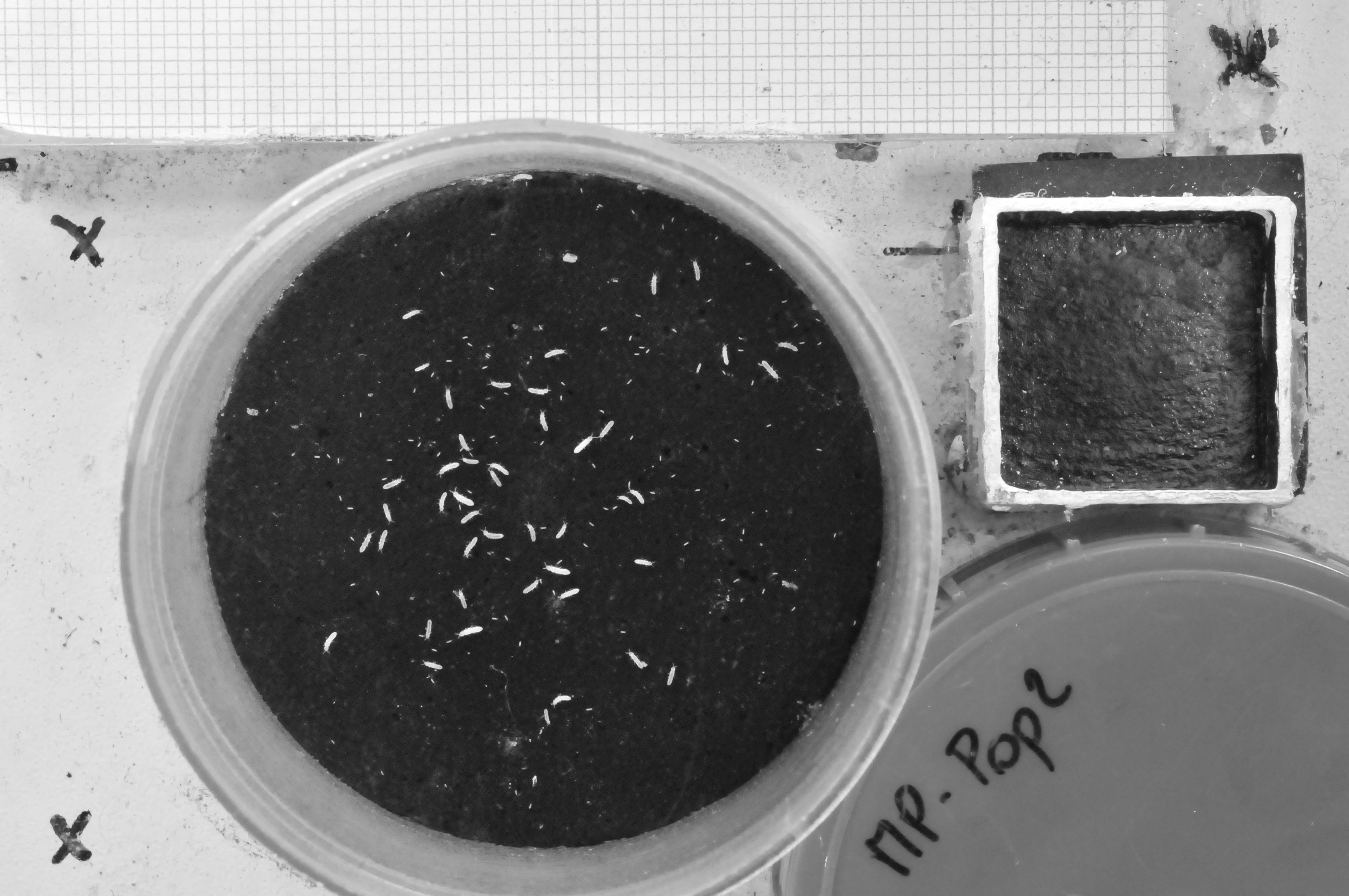

Supplement: File S3 — Two sets of pictures of populations of collembolans that can be used as examples to try the plugin. (ZIP) [file pone.0064387.s006.zip › Picture_set_example1/PR_2011-06-05_181727.jpg]

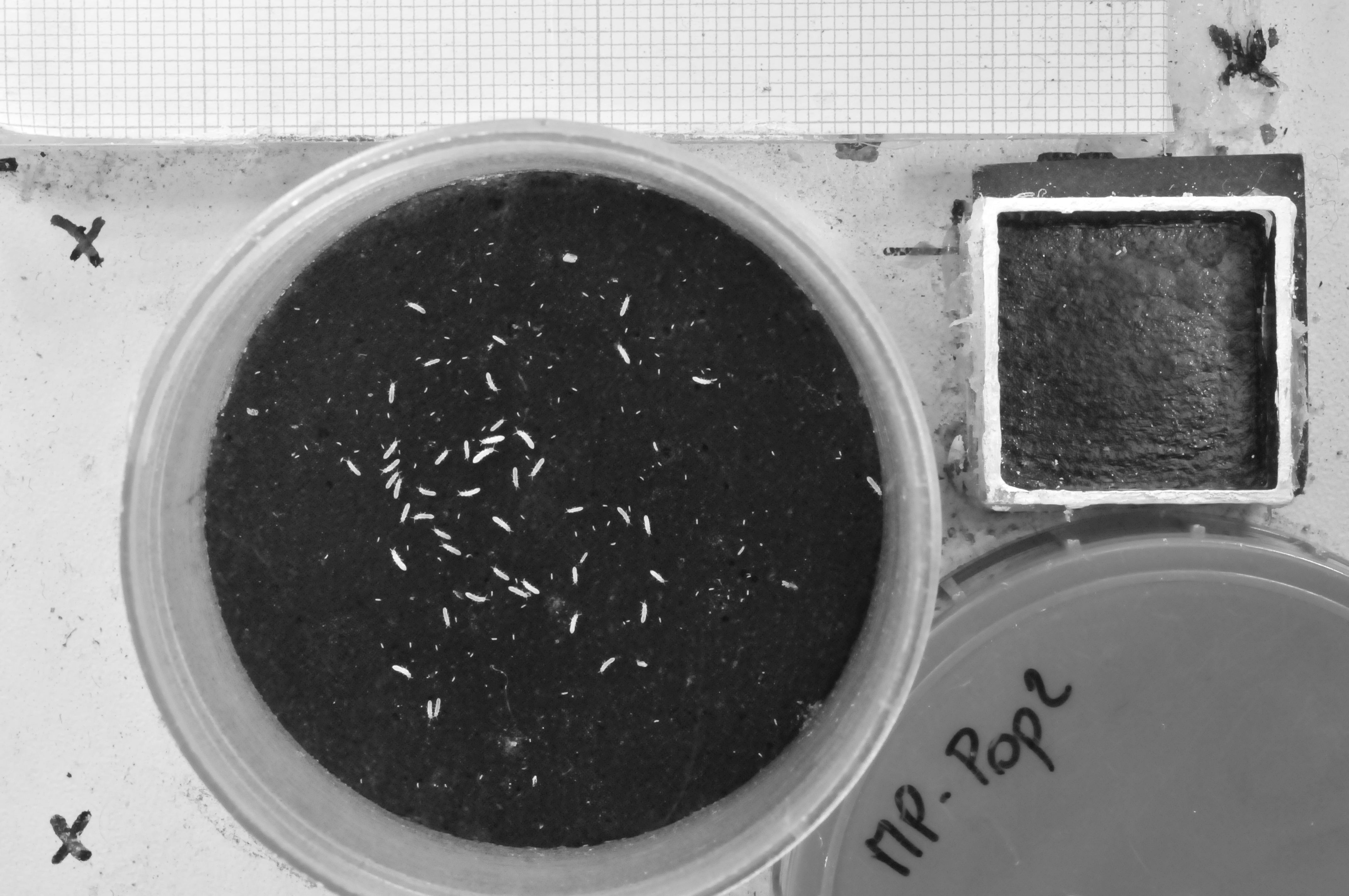

Supplement: File S3 — Two sets of pictures of populations of collembolans that can be used as examples to try the plugin. (ZIP) [file pone.0064387.s006.zip › Picture_set_example1/PR_2011-06-05_181730.jpg]

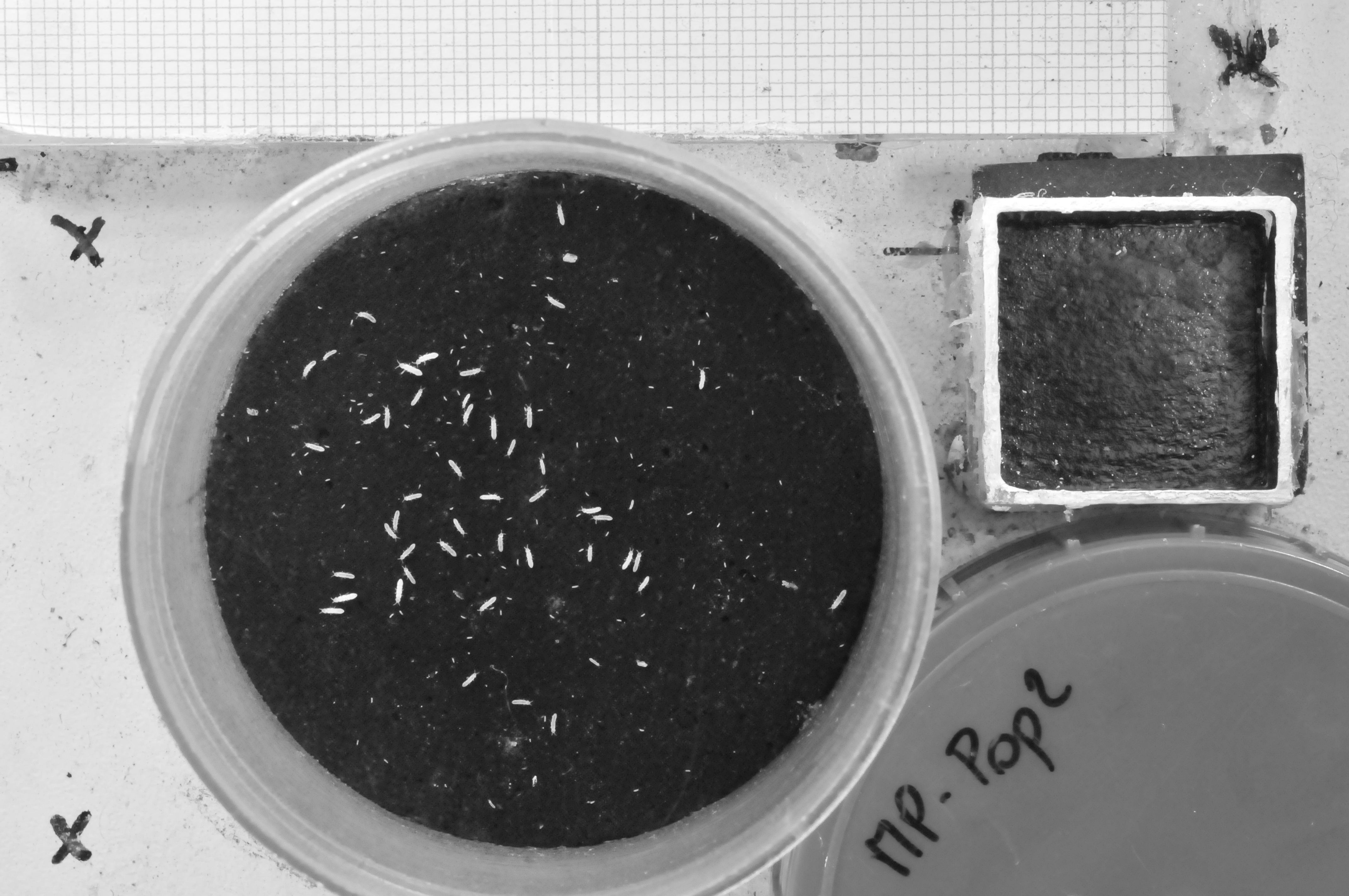

Supplement: File S3 — Two sets of pictures of populations of collembolans that can be used as examples to try the plugin. (ZIP) [file pone.0064387.s006.zip › Picture_set_example1/PR_2011-06-05_181733.jpg]

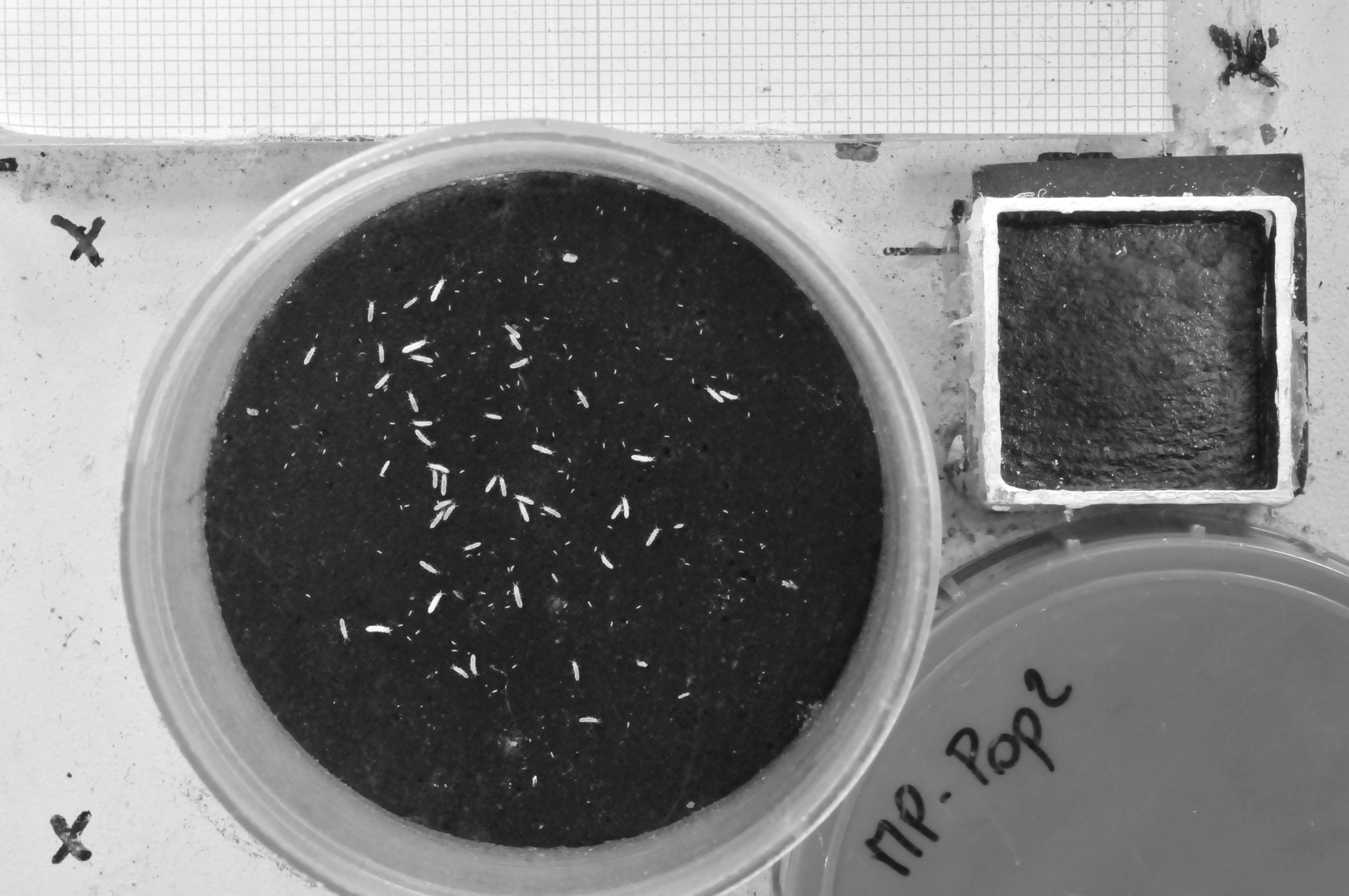

Supplement: File S3 — Two sets of pictures of populations of collembolans that can be used as examples to try the plugin. (ZIP) [file pone.0064387.s006.zip › Picture_set_example1/PR_2011-06-05_181736.jpg]

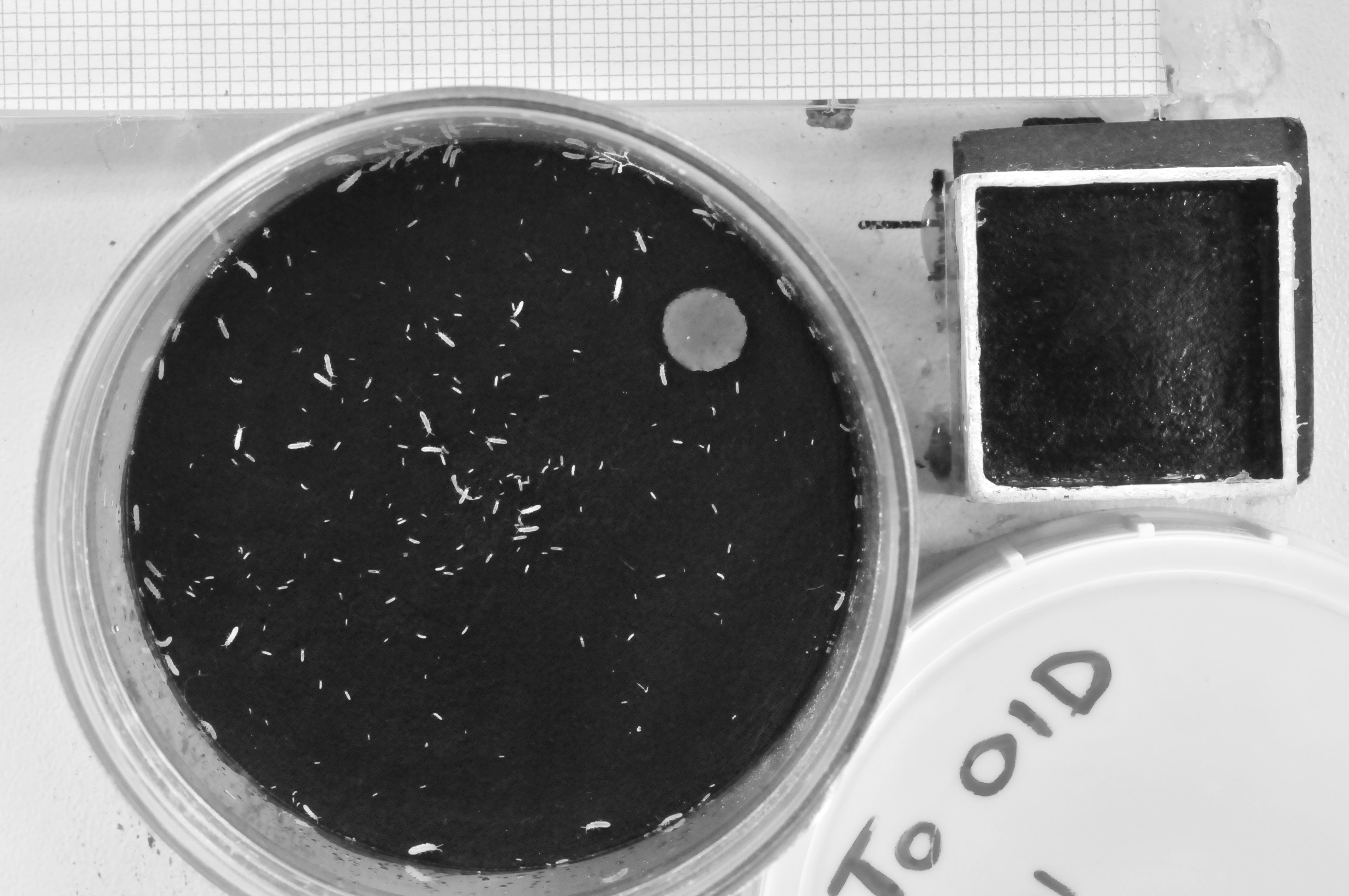

Supplement: File S3 — Two sets of pictures of populations of collembolans that can be used as examples to try the plugin. (ZIP) [file pone.0064387.s006.zip › Picture_set_example2/XX_2011-02-15_122831.JPG]

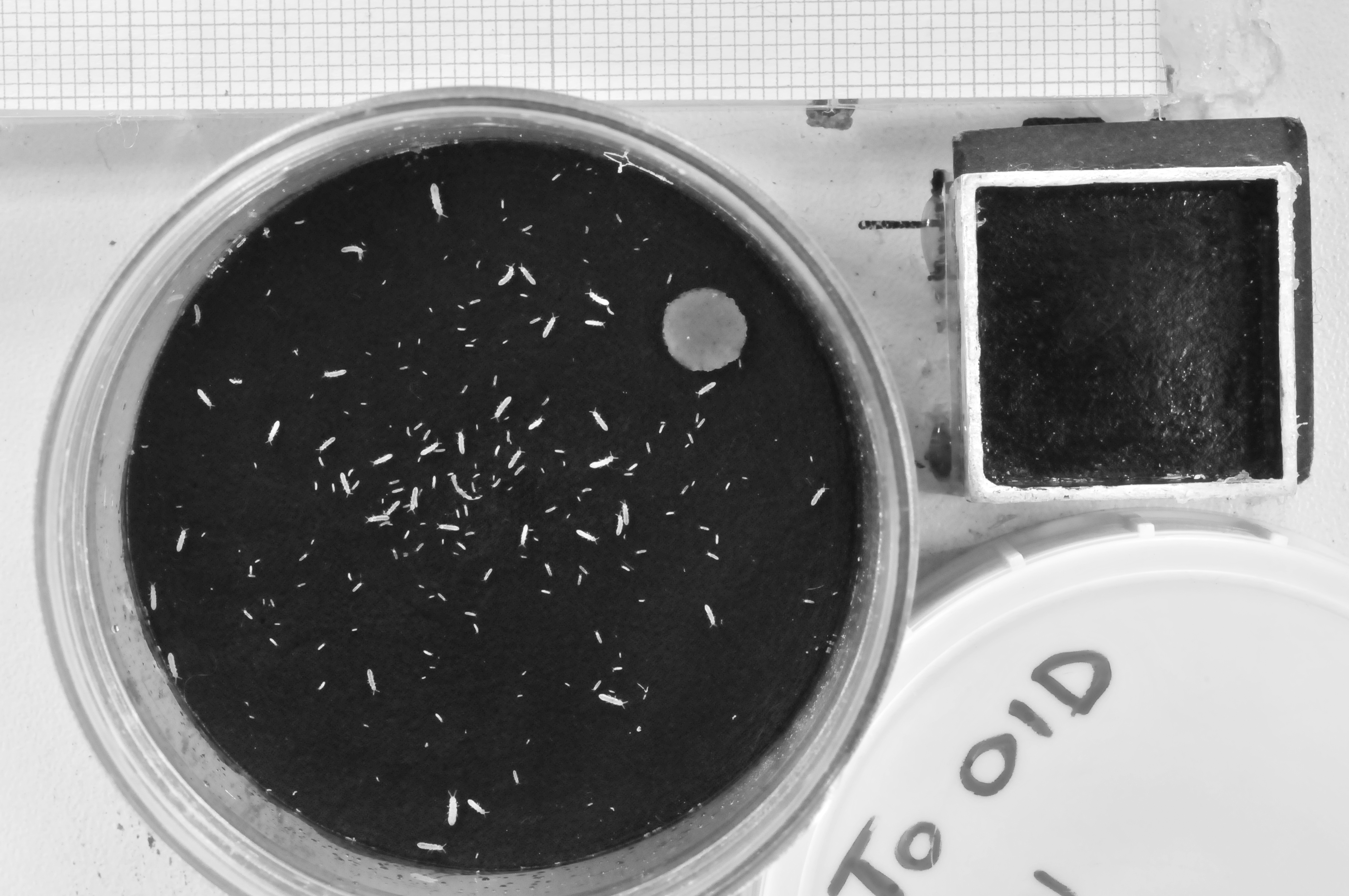

Supplement: File S3 — Two sets of pictures of populations of collembolans that can be used as examples to try the plugin. (ZIP) [file pone.0064387.s006.zip › Picture_set_example2/XX_2011-02-15_122834.JPG]

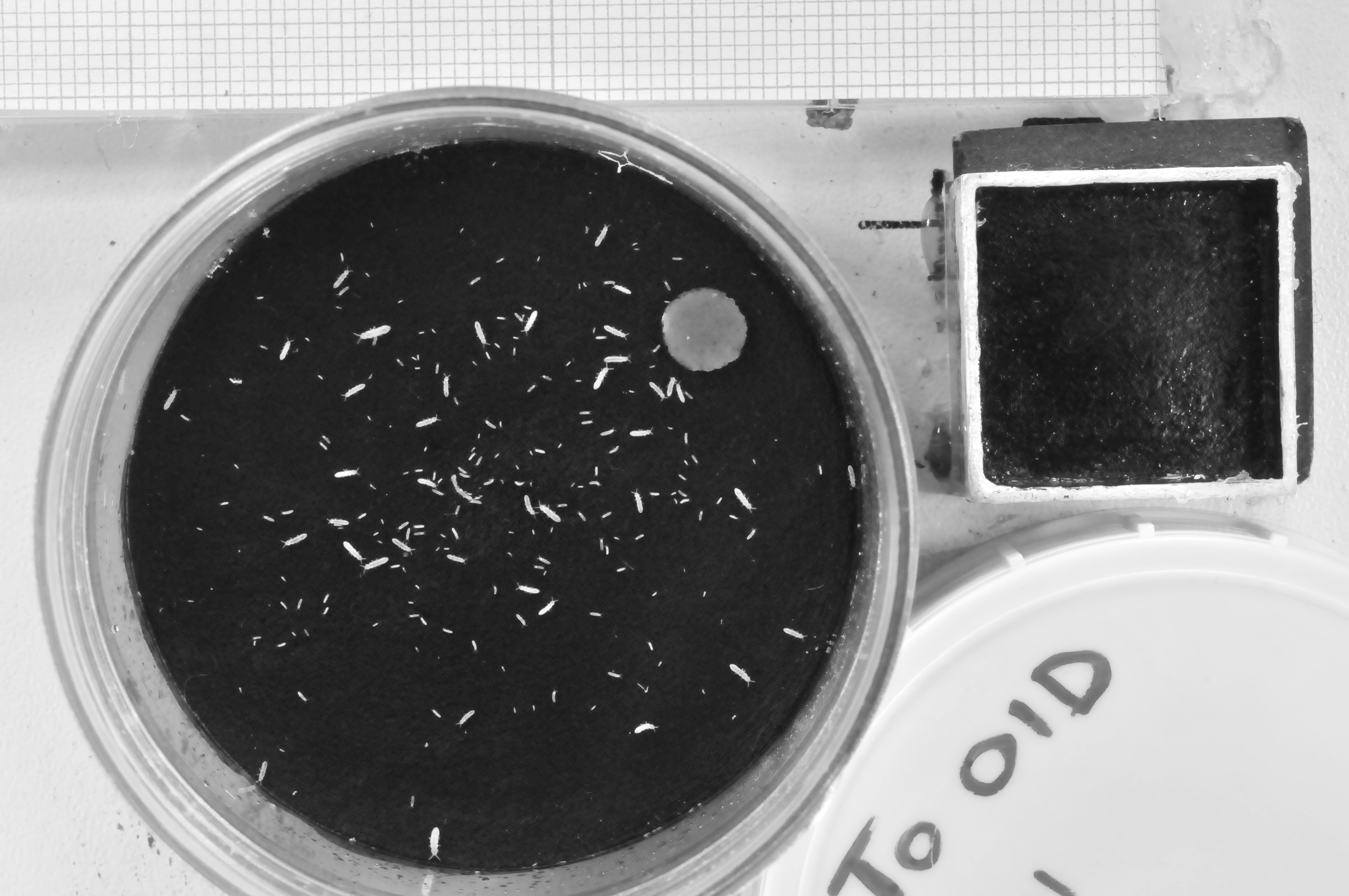

Supplement: File S3 — Two sets of pictures of populations of collembolans that can be used as examples to try the plugin. (ZIP) [file pone.0064387.s006.zip › Picture_set_example2/XX_2011-02-15_122837.JPG]

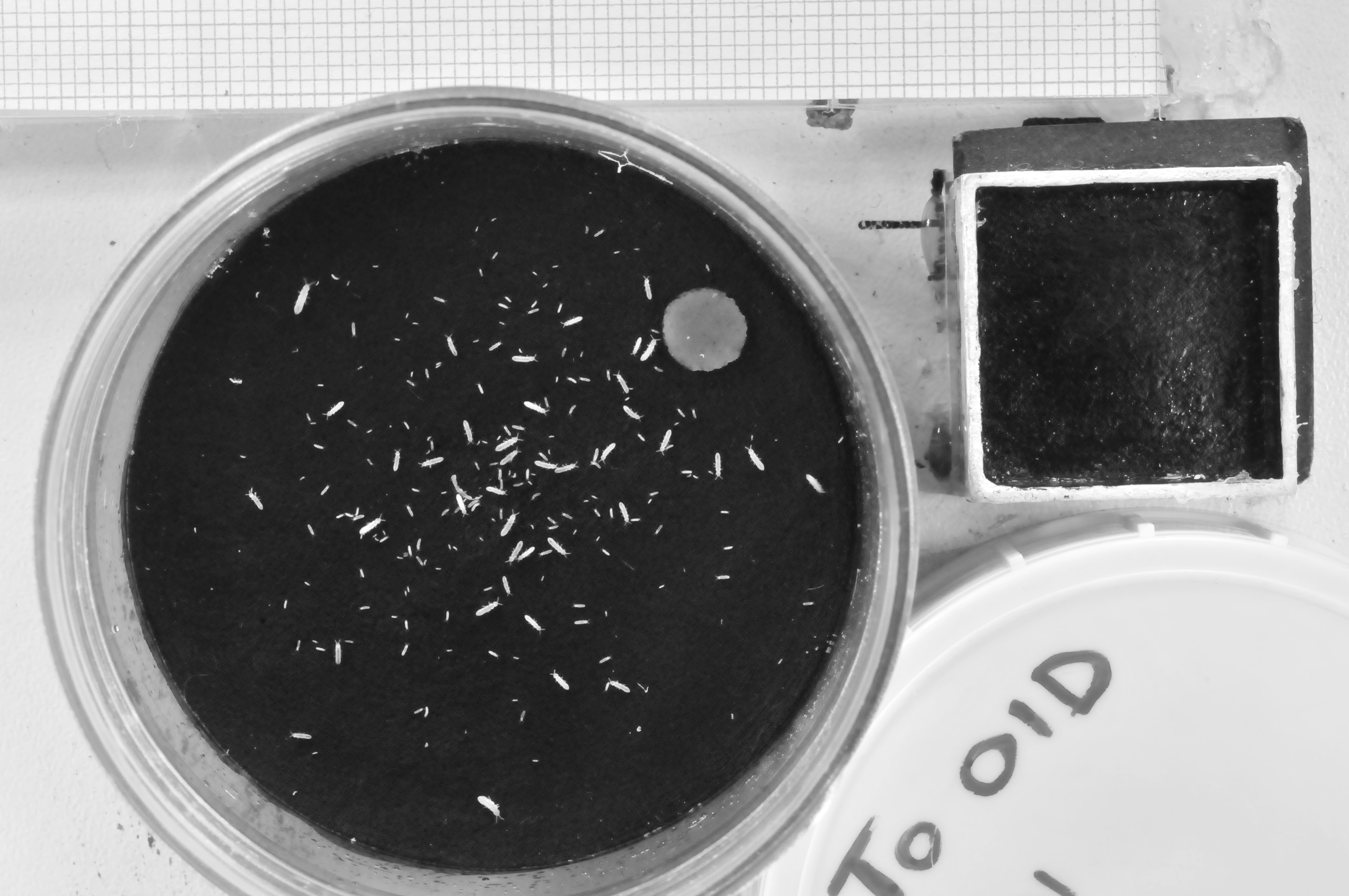

Supplement: File S3 — Two sets of pictures of populations of collembolans that can be used as examples to try the plugin. (ZIP) [file pone.0064387.s006.zip › Picture_set_example2/XX_2011-02-15_122840.JPG]

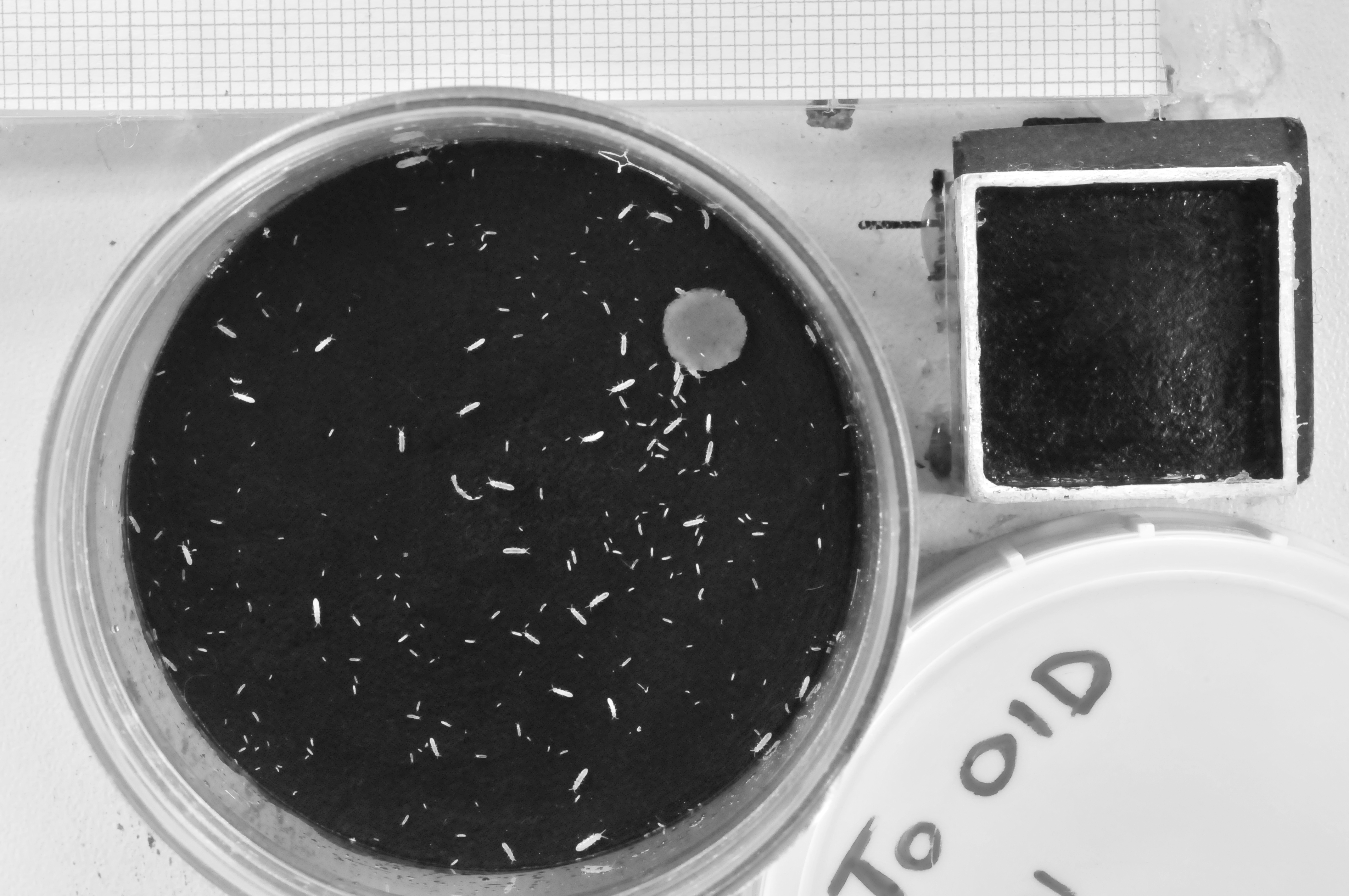

Supplement: File S3 — Two sets of pictures of populations of collembolans that can be used as examples to try the plugin. (ZIP) [file pone.0064387.s006.zip › Picture_set_example2/XX_2011-02-15_122843.JPG]
