# Supplementary material for: An Automated Image Analysis System to Measure and Count Organisms in Laboratory Microcosms
Source: PLoS One. 2013 May 29;8(5):e64387. doi: 10.1371/journal.pone.0064387 (PMC3667193; doi:10.1371/journal.pone.0064387)
Supplement: Figure S2 — Plugin specification windows. (a) On this window one is asked to specify the variables described in Table S1 in File S1. If selected, the automatic scaling specification window (b) and the region of interest (roi) selection specifications window (c) will open successively. (PDF) [file pone.0064387.s002.pdf]

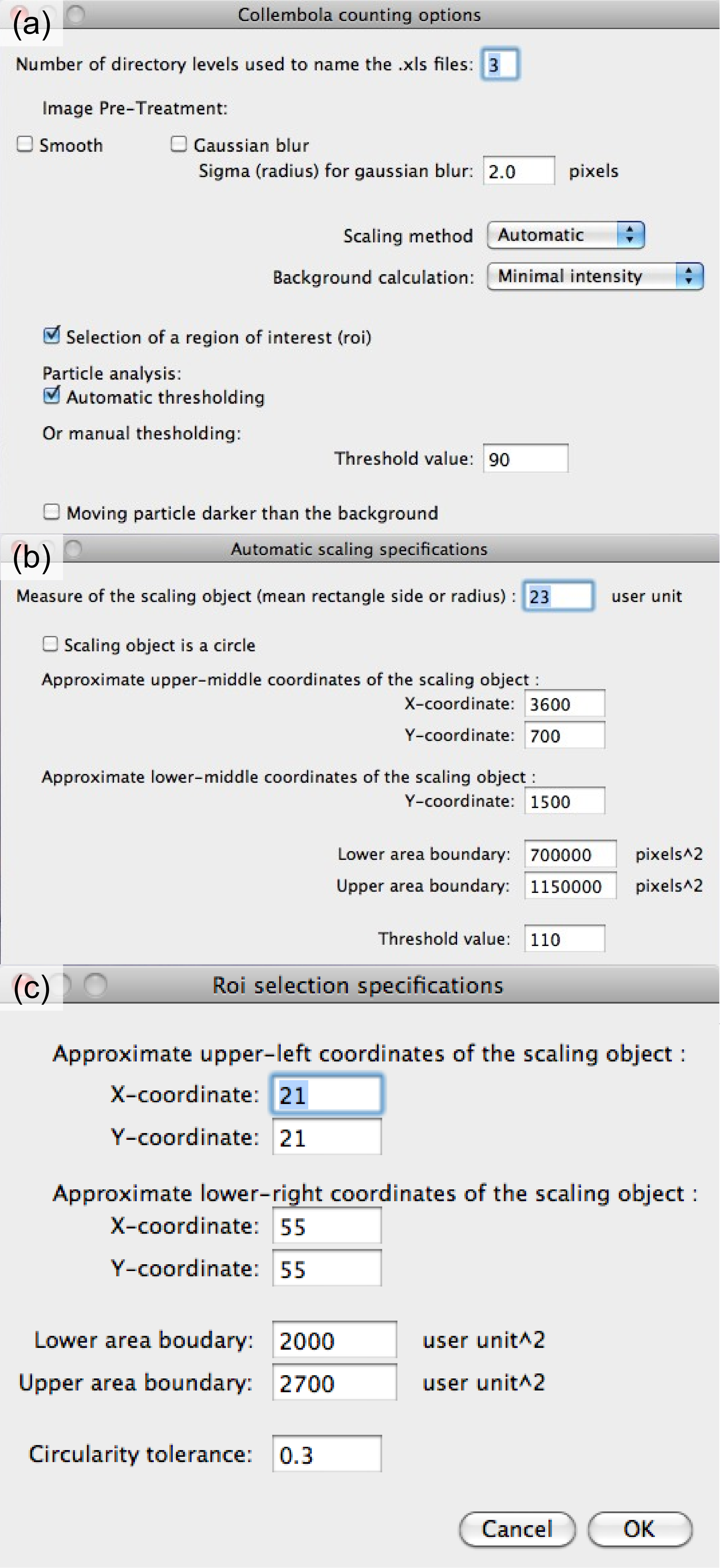
(a)
Collembola counting options
Number of directory levels used to name the .xls files: 3
Image Pre-Treatment:
Smooth
Gaussian blur
Sigma (radius) for gaussian blur: 2.0 pixels
Scaling method Automatic
Background calculation: Minimal intensity
Selection of a region of interest (roi)
Particle analysis:
Automatic thresholding
Or manual thesholding:
Threshold value: 90
Moving particle darker than the background
(b)
Automatic scaling specifications
Measure of the scaling object (mean rectangle side or radius) : 23 user unit
Scaling object is a circle
Approximate upper-middle coordinates of the scaling object :
X-coordinate: 3600
Y-coordinate: 700
Approximate lower-middle coordinates of the scaling object :
Y-coordinate: 1500
Lower area boundary: 700000 pixels^2
Upper area boundary: 1150000 pixels^2
Threshold value: 110
(c)
Roi selection specifications
Approximate upper-left coordinates of the scaling object :
X-coordinate: 21
Y-coordinate: 21
Approximate lower-right coordinates of the scaling object :
X-coordinate: 55
Y-coordinate: 55
Lower area boudary: 2000 user unit^2
Upper area boundary: 2700 user unit^2
Circularity tolerance: 0.3
Cancel
OK
