# Supplementary material for: An Automated Image Analysis System to Measure and Count Organisms in Laboratory Microcosms
Source: PLoS One. 2013 May 29;8(5):e64387. doi: 10.1371/journal.pone.0064387 (PMC3667193; doi:10.1371/journal.pone.0064387)
Supplement: File S1 — Some explanations to help understand how the imageJ plugin works and how it can be customised. Table S1 in this file summarises the different plugin’s options. (DOC) [file pone.0064387.s004.doc]

Supporting Information: An automated image analysis system to measure and count organisms in laboratory microcosms

François Mallard, Vincent Le Bourlot and Thomas Tully

�gBatch population sensor�h, an ImageJ plugin to automate the analysis of digital pictures of laboratory microcosms.

The plugin BP_sensor.java automates the successive steps of the analysis and recursively analyses multiple sets of images, producing rapidly measurements from a large number of replicated microcosms.
In order to run the ImageJ plugin "BP_sensor" the following files have to be saved in the ImageJ plugins folder or a sub-folder thereof : BP_sensor.java and Wait_For_User.java.
More information on the plugin "Wait for user" can be found on the ImageJ Documentation Wiki : http://imagejdocu.tudor.lu/doku.php?id=plugin:utilities:wait_for_user:start.
Once the files saved, compile first "Wait_For_User.java" and then "BP_sensor.java" with "Compile and Run". Restart ImageJ, you can now launch the plugin and follow the instructions. To perform the analysis on the picture set example provided you need to save them in a given directory that you will be asked to specify when running the plugin.
As described in the main text, the core of the image processing consists, for each population, in analysing several pictures of the same microcosm and in comparing these pictures in order to remove the still background for an enhanced image segmentation. This process is embedded into other functions to batch process many stacks of images, scale the measurements, detect the region of interest in the image where the counting has to be run, adjust the particle detection threshold and automatically export the data into spreadsheet files (Table S1).
The first step is to take successive pictures (ideally three to five) of the microcosms under study in stable conditions using a camera stand, a remote shutter release and constant lighting conditions. The different sets of pictures are to be sorted in a file tree as shown in Figure S1. When the plugin is started, one has to specify the location of this file tree and the folder in which the produced data files will be saved. Then several optional functions can be activated (Figure S2). These functions are described in Table S1. Note that since most of them are written as sub-functions in our java code, they can be easily modified and customised for other setups. The default values of the input windows can also be changed in the java code. After each stack measurement, the results of the particle analysis are saved in a distinct .xls files (Figure S1) named after the directory path that contains the analysed pictures. It is possible in the main option window to specify the number of directory levels that will be included in the output file names. Note that for the plugin to run fine, one must not use the space character in the files or folders' names.
As an example, we provide in the supporting material a set of pictures that can be analysed with our plugin using the default values. The main steps are illustrated in Figure S3: the background picture is first constructed (Figure S3 b) by comparing the different images in the stack (Figure S3 a), keeping only the minimal values (darker) for each pixel. This background picture is then removed from the different images of the original stack. It creates a new stack where only the moving particles remain (Figure S3 d). In this example, the background image is also used to scale the measurements. A black square on the top right of the picture (arrow 2) is selected and measured to automatically convert in mm the future measurements. It is also possible to select a reference distance (arrow 1) to manually adjust the scale. The plugin will then detect the boundary of the rearing box (Figure S3 b, arrow 3), and retrieve on the new stack of images this selected region of interest (Figure S3 d). Within this boundary, an automatic threshold is applied and the particles are measured and counted (Figure S3 d) The background removal is an essential step that makes possible the automation of the thresholding. As shown in Figure S3, most of the substrate heterogeneity is removed. Motionless particles or dead organisms are also excluded from the automatic census (Figure S3, arrows 4 and 5). Without this crucial step, a batch processing of large amounts of replicated populations would not be possible and a threshold would have to be adjusted manually.
Some parts of the provided java code can also be used separately: the automatic scaling procedure can be used to speed up recursive manual size measurements on pictures shot at various heights (for example, in field experiments without a camera stand). One just has to add on each picture, at a relatively constant position, a contrasted area that can be used for scaling.

Table S1: Overview of the plugin�fs options.
Pre-treatment	Optional smoothing of the pictures before launching the particle analyses. Two methods are available: the smooth function of ImageJ (average pixel value in a 3x3 square) or a gaussian blur.	
Scaling	A pixel-millimetre (or another unit) conversion ratio is estimated and enables ImageJ to directly measure particles in millimetres. Two methods are proposed, manual and automatic.
–	manual: the user is asked to measure a known area and to report its real value (mm2) and its surface in number of pixels.
automatic : the plugin will automatically detect and measure a contrasted black rectangle or circle of known size used as reference. An input window will open to specify (1) the radius length for a circle, the side length for a square or the mean length of the short and long sides for a rectangle (in mm for instance). The user will also be asked to specify the approximate position (pixel coordinates) of the scaling object on the picture as well as the two extreme values of an interval which is sure to contain this reference area. Theses parameters will be used by the plugin to search, detect, select and measure this reference area and to scale the subsequent measurements.	
Background calculation	Three calculation methods are proposed to generate the background picture: minimal (darker), average or median value for this �gz-projection�h of the original stack. Note that average or median are preferred to buffer lighting heterogeneity between the images in the stack, but they usually require more images to perform well.	
Selection of a region of interest	This option is useful if one wants to ensure that the program will only analyse the particles that are within a specific region of interest (roi) of the picture. If selected, a new input window will open to specify its approximate position (pixel coordinates in the image) as well as the two extreme values of an interval which is sure to contain the surface area for this roi. The plugin will try to find the larger black region within this interval size that maximises the circularity. The user is also asked to supply a minimal circularity value. If no roi is found above this value, the user will later be asked to manually select the roi.	
Particle analysis threshold	The user can choose an automatic thresholding method, "Intermodes", that assumes a bimodal distribution and fix the threshold at the mean value of the two local extrema. Alternatively, a fixed threshold value can be imposed.	
Moving particles darker than their substrate	The plugin can invert the pictures when loading the stack. All the steps will then be performed the same way. This can be useful if one has dark particles moving on a lighter background.	
